# Phonological processing of ignored distractor pictures, an fMRI investigation

**DOI:** 10.1186/1471-2202-9-20

**Published:** 2008-02-11

**Authors:** Mart Bles, Bernadette M Jansma

**Affiliations:** 1 Department of Cognitive Neuroscience, Faculty of Psychology, Maastricht University, Maastricht, the Netherlands; 2 Bernstein Center for Computational Neuroscience Berlin, Charité – Universitätsmedizin, Berlin, Germany

## Abstract

**Background:**

Neuroimaging studies of attention often focus on interactions between stimulus representations and top-down selection mechanisms in visual cortex. Less is known about the neural representation of distractor stimuli beyond visual areas, and the interactions between stimuli in linguistic processing areas. In the present study, participants viewed simultaneously presented line drawings at peripheral locations, while in the MRI scanner. The names of the objects depicted in these pictures were either phonologically related (i.e. shared the same consonant-vowel onset construction), or unrelated. Attention was directed either at the linguistic properties of one of these pictures, or at the fixation point (i.e. away from the pictures).

**Results:**

Phonological representations of unattended pictures could be detected in the posterior superior temporal gyrus, the inferior frontal gyrus, and the insula.

**Conclusion:**

Under some circumstances, the name of ignored distractor pictures is retrieved by linguistic areas. This implies that selective attention to a specific location does not completely filter out the representations of distractor stimuli at early perceptual stages.

## Background

A typical property of human perception is its selective nature. Only a limited amount of information from our environment reaches our awareness, indicating that our brain selects some objects for further processing, while ignoring others. It is generally assumed that the purpose of this selection is the more efficient processing of selected stimuli than would be the case if all stimuli were processed simultaneously. Most attention research has therefore focused on the enhanced processing of attended stimuli, showing that attended stimuli are easier to detect and to identify. Considerably less is known, however, about the fate of ignored stimuli. The main target of this study was to investigate whether information about ignored distractor stimuli becomes available in brain areas beyond typical object processing areas. Specifically, we investigated whether phonological areas activate the phonological code of ignored distractors. Accordingly, we gain insight in selection mechanisms both at a visual and a phonological level.

It is generally assumed that the necessity for selection depends on the limited processing capacity of our sensory systems, which prevents each stimulus to be represented to the full extent. According to the influential 'biased competition' approach, simultaneously presented stimuli compete for neural representation and behavioural control, and engage in suppressive interactions (for an overview, see [1-4]). For example, Kastner et al. [5-8] have shown that when participants viewed simultaneously presented stimuli, BOLD-signal change was reduced compared to sequential viewing of the same stimuli (where no suppressive interactions could take place). These results support the view that simultaneously presented stimuli suppress each others' neural representation, and thereby reduce the magnitude of the BOLD response. However, competition can be biased in favour of a stimulus through bottom-up or top-down mechanisms. In such a case, this stimulus will be more successful in suppressing the representation of the others, and its neural representation will take precedence over that of the others. Indeed, neural suppression effects diminished when one of the stimuli was very salient [9] or when one of the stimuli was attended [5,6,8,10], showing that these stimuli had succeeded in overcoming the suppressive effects of the distractor stimuli.

An issue that has occupied researchers for decades is what happens to the neural representation of the distractor stimuli. Is the representation of these stimuli abolished, or do (attenuated) representations exist beyond the areas in which competition takes place? According to early-selection accounts, attention selects certain stimuli from the environment at the perceptual level, while ignoring irrelevant distractor stimuli. Since these distractor stimuli are filtered out at a perceptual level, the brain is unaware of their identity, and participants are typically unable to report many characteristics about these stimuli [11,12]. Proponents of late selection theories, instead, argue that all stimuli are perceptually processed and identified, but that selection takes place at a higher-order level like working-memory representation or response selection. In this case, attention serves as a filter for determining which perceived objects reach awareness [13-15]. For example, when a judgment needs to be made about the identity of a target letter at fixation, responses are generally faster when ignored flanker items are congruent with the target letter than when they are incongruent, indicating that the flankers are identified [15,16].

Recently, some authors have tried to resolve discrepancies between these views and integrate early and late selection mechanisms [17-19]. These frameworks postulate that attention can filter distractor information either at an early or at a late level, depending on the available resources and task requirements. Typically, attention acts at the perceptual level in cases where the perceptual system is overloaded and a selection needs to be made which information is further processed. When such selections are not necessary, however, irrelevant stimuli will also be processed by sensory areas, but attention prevents them from being represented in working memory, thereby blocking awareness of these stimuli. According to these theories, identification of irrelevant stimuli occurs in cases in which resource demands are low.

Neuroimaging techniques provide a good method of choice to trace the processing of ignored distractors within the brain, since they enable the mapping of influences of stimuli throughout several cortical areas (with fMRI) *without *the need for a participant's overt response. Some investigators have indeed detected activation traces associated with ignored distractors beyond early visual areas. For example, some studies have shown that the decreased BOLD response typically associated with stimulus repetition also occurs with repetition of unattended stimuli (albeit smaller) [20-22]. In line with load theory [19], these effects disappeared when task demands were high [20,23,24], suggesting that ignored stimuli are involuntarily processed in case task demands do not require all available resources. Ruz et al. [25] observed that ERPs differentiated between words and non-words even though attention was highly engaged by another task (but see [26]). According to the authors, these results indicate that, at least for highly learned stimuli such as letter strings, some processing resources are automatically dedicated even though participants are engaged in an attentionally demanding task.

The goal of the present study was to investigate whether cortical areas beyond visual areas engage in processing ignored stimuli. Specifically, we investigated whether phonological relatedness between names of different pictures influences processing in regions that process meaning and phonology. Finding such a result would imply that, despite being ignored, distractor pictures are represented in areas beyond visual cortex. The question then would be whether this effect was dependent on the location of attention and the task the participant is performing.

Previous research suggests that the activation of semantic features of stimuli may occur automatically, even for ignored stimuli. In paradigms like picture-picture interference (PPI) or negative priming (NP), participants view simultaneously presented stimuli, one of which is to be named and the other to be ignored. Typically, they are slower in naming target pictures if the pictures within a trial are semantically related, compared to unrelated trials (PPI) [27] (but see [28]), or if the current target picture was ignored in the previous trial (NP) [29] (see [30,31] for a review). Importantly, NP can even be observed if the ignored prime is a picture and the target is a word [32], showing that the processing of the ignored picture involves at least the identification of semantic features. Further support for a semantic locus of this effect was provided by the observation that the left anterolateral temporal cortex, which has been associated with processing of semantic knowledge [33], is activated in trials in which NP was observed [34].

Although phonological activation of ignored distractor *words *is fairly well-documented [35,36], activation of phonological properties of ignored distractor *pictures *has only recently been observed. In PPI studies, faster reaction times have been observed when the ignored picture was phonologically related to the target (e.g. "DOG" – "DOLL") than when they were unrelated (e.g. "DOG" – "BED") [37-39]. This finding implies that the phonological code of the distractor picture was activated, even though it was ignored.

**Figure 1:**
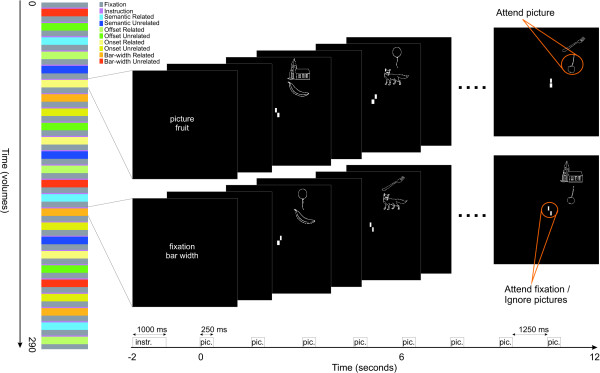
**Experimental design**. Each participant performed four functional runs, including 24 task blocks each. These included three linguistic tasks (onset monitoring, offset monitoring, semantic categorisation) in which the lower picture was attended. In the perceptual bar-width discrimination task, the pictures were ignored. Blocks were either phonologically related (e.g. "KERS" – "KERK" ('cherry' – 'church')) or unrelated (e.g. "KERS" – "VORK" ('cherry' – 'fork')), leading to a 4 (task) * 2 (relatedness) design, which was repeated 3 times per run in a semi-randomised order.

The present study was aimed at detecting phonological-code activation for ignored distractor pictures with fMRI. In order to investigate this, a modified version of a paradigm that has proven successful in visual attention research [10] was employed. Since we were also interested in whether linguistic processes would influence processing in visual cortex, the pictures were presented at separate peripheral locations. Sets of pictures were presented simultaneously, while participants performed one of three linguistic tasks in which they attended one of the pictures, or a central task at fixation (i.e. ignore the picture). Of the three attended tasks, two focussed on phonological processing (onset monitoring, offset monitoring), and one focused on meaning (semantic-category judgement). The fixation task aimed to withdraw attention from the pictures and involved visual line discrimination. Whereas this design was previously employed to measure suppression effects between abstract, colourful stimuli in visual cortex, the present adaptation entails the use of concrete black-and-white line drawings. By assigning meaning to pictures, it becomes possible to measure haemodynamic responses to the pictures in cortical areas associated with semantic and phonological processing. Moreover, we manipulated the amount of phonological overlap between the names of the simultaneously presented pictures. In the 'related' condition, the names of the pictures consisted of at least an identical first consonant-vowel construction, e.g. a picture of a "VOS" (fox) and a picture of a "VORK" (fork), see figure 1). In the 'unrelated' condition, there was no such phonological overlap (a picture of a "VOS" (fox) and a picture of a "HARK" (rake)). The underlying rationale is that if phonological processing areas respond differently to related sets than to unrelated sets, the phonological properties of both target and distractor pictures must have been identified. This would imply that, at least under these circumstances, even though visual attention is highly focused on a single object or location, attention attenuates the processing of unattended objects, rather than filter them out completely.

We expected to observe effects of phonological relatedness mainly in cortical areas involved with generating the pictures' names. Specifically, we were interested in the left posterior superior temporal gyrus (STG) and the left anterior insula, which are presumably involved in phonological code retrieval [40,41], and in the left inferior frontal gyrus (IFG), believed to be involved in syllabification and phonological processing [40,42-44]. The left posterior STG (often referred to as Wernicke's area), has been observed to show decreased activation in a picture-word interference paradigm when pictures were presented with phonologically related distractor words, compared to unrelated words [45]. Assuming that the lower BOLD response is linked to a reduced amount of neural processing, this observation is in line with the observation that response times are generally faster in phonologically related trials [46-48].

In addition to the question whether relatedness effects would be observed in phonological processing areas, we were interested in the interaction of this effect with the task a participant is performing. In particular, whereas it is quite straightforward to anticipate relatedness effects in the two phonological tasks, some authors have suggested that the level of representation of stimuli depends on the task demands [30,49], suggesting that no phonological relatedness effects should be observed during non-phonological semantic categorisation, or during a visual line comparison task. In the onset-monitoring task, participants were asked to judge whether the name of the picture closest to the fixation point started with a certain letter. In order to carry out this task, participants had to internally generate at least the first part of the name of the picture. This task most directly aimed to test effects of distractor relatedness, as the experimental relatedness between target and distractor was realised at this onset-letter position. The offset-monitoring task was similar, but involved the decision whether the picture name ended with a certain letter. In order to perform this task, participants had to generate the entire name of the target, including the onset of the name, in which overlap exists with the onset of the distractor picture's name in related trials. The semantic task involved a category judgment in which participants responded to pictures belonging to a certain category (e.g. mammals, musical instruments). No explicit generation of the pictures' names was required for successful performance on this task. The control task (ignore the picture) involved an attentionally demanding line-comparison task at fixation. This condition served as a visual control, with exactly the same physical stimulation parameters as the other tasks, but without participants attending one of the pictures.

In this design, areas that are involved in phonological encoding, such as the STG and the posterior IFG, were expected to be activated stronger in the phonological tasks than in the semantic or control task. The question was whether within those areas effects of phonological relatedness could be observed, indicating that the names of the ignored pictures are activated as well. Furthermore, we investigated whether those effects were also visible in the same areas in non-phonological (i.e. semantic-category judgment and line comparison) tasks, indicating that name generation occurs even when phonological processing is not required. Finally, we were interested in whether phonological relatedness effects would be observed in areas involved in semantic processing, such as the left prefrontal cortex and the left middle temporal gyrus (see [50] for a review), and visual processing, (see [51]). This requires exact localisation of early visual areas and the Lateral Occipital Complex, involved in object recognition [52,53]. Therefore, standard retinotopy and LOC localiser runs were included in the experiment. Finding effects of phonological relatedness in these areas would imply that, presumably through top-down biasing signals, phonological features can influence picture processing in non-phonological areas.

## Results

### Behavioural data

Reaction times associated with each condition are reported in table 1. A repeated measures GLM with the factors task and relatedness revealed a significant main effect of task on reaction time (F (3, 39) = 41.71, p < .001). Post-hoc analysis revealed that reaction times were fastest in response to the semantic task (615 ms), followed by the control task (651 ms), the onset (717 ms) and offset monitoring task (773 ms), respectively. The difference between each of the tasks was significant (all p-values < .04, Bonferroni-corrected). No effect of relatedness was observed (F (1, 13) = .05, p = .82), nor was there any interaction between relatedness and task (F (3, 39) = .47, p = .67). Error rates were generally low (< 2%) and did not differ across conditions.

**Table 1:** Mean reaction times in milliseconds (standard errors in parentheses)

	**Related**	**Unrelated**
**Semantic**	615 (20)	614 (18)
**Onset monitoring**	711 (18)	722 (19)
**Offset monitoring**	776 (19)	769 (19)
**Control**	649 (19)	652 (18)

### Fixed effects analysis

**Figure 2:**
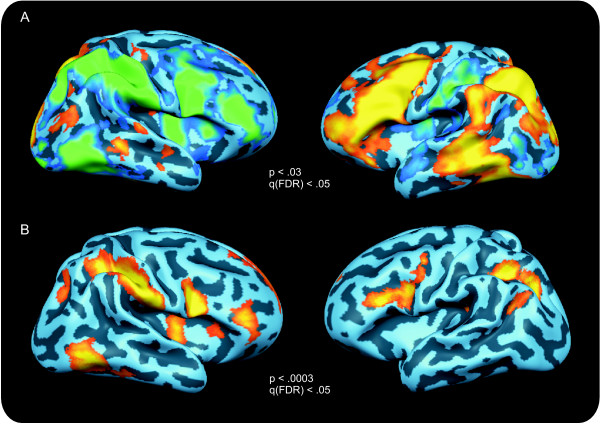
**Task effects**. The fixed-effects group analysis (bar-width discrimination vs. linguistic tasks) revealed a strong, task-dependent lateralisation visible in figure 2A. The bar-width discrimination task (blue-to-green scale) mainly activated a large-scale right-hemispheric network (2A, left panel), whereas the linguistic tasks (phonological onset or offset monitoring and semantic categorisation, orange-to-yellow scale) activated a left-hemispheric network (2A, right panel). A random effects ANCOVA model showed that the main effect of task was most reliable in the right-hemispheric network, and in left-hemispheric frontal and parietal areas (2B). All maps are thresholded at q (FDR) < .05.

Compared to baseline periods, the tasks reliably activated an extensive bilateral network of areas including parietal areas, occipital areas including early visual areas, inferior temporal areas including large parts of the fusiform gyrus, the posterior parts of the STS and the superior temporal gyrus (STG), the insulae, bilateral IFG and middle frontal gyri (MFG), and the medial frontal gyri. Within these areas, an extensive lateralisation of tasks was observed (see figure 2A). The linguistic tasks (onset and offset monitoring, and the categorisation task) mainly activated left IFG and MFG, the posterior parts of the STS/STG, parietal and inferior temporal areas, and early visual areas, compared to the control task. This control task, on the other hand, mainly activated right IFG and MFG, parietal and inferior temporal areas and the right insula. Phonological tasks activated the left inferior and superior parietal lobule, left insula, left MFG and IFG (BA9/BA44/BA45) and the medial frontal gyrus more strongly than the semantic task. The reverse was true for the anterior IFG (BA47) and large portions of the middle temporal gyrus.

The (Unrelated > Related) contrast revealed some scattered areas throughout bilateral inferior temporal areas, the posterior parts of the STS/STG and parietal areas. However, inspection of the time courses within these areas revealed that most of these areas were only slightly activated during these tasks and that the differences between relatedness conditions seemed mainly caused by baseline differences between conditions. This was confirmed by a conjunction contrast (Unrelated > Related) ∩ (Task > Baseline) which revealed a main effect of relatedness in right inferior temporal areas and a posterior area of the right MTG.

Due to the expected interaction between relatedness and task, we investigated relatedness effects in each task separately. In the onset task, the (Unrelated Onset > Related Onset) ∩ (Onset > Baseline) contrast revealed only the middle part of the right central sulcus.

**Figure 3:**
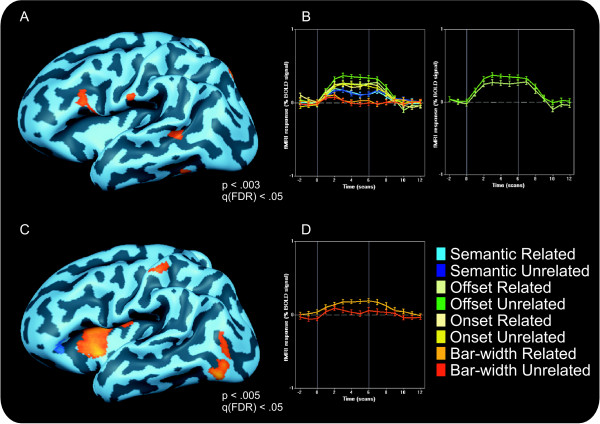
**Relatedness effects**. A fixed-effects group analysis revealed that in the offset task, related blocks led to decreased activity compared to unrelated blocks in the left IFG and STS/STG (3A, offset unrelated > offset related). The time course of this effect in the IFG is depicted in fig. 3B. In the bar-width discrimination task, related blocks led to *increased *activity compared to unrelated blocks. This effect, revealed by the control related > control unrelated contrast shown in fig. 3C was most apparent in the left insula. The time course of the insular activation is depicted in fig. 3D. All contrast maps are thresholded at q (FDR) < .05.

For the offset task, the contrast (Unrelated Offset > Related Offset) ∩ (Offset > Baseline) revealed the left IFG (BA44) and an inferior part of the MFG (BA9), the posterior part of the STS/STG, bilateral precunei and bilateral inferior temporal cortices, the right cingulated gyrus and the inferior part of the left post-central gyrus (see figure 3A and 3B).

No effects of relatedness were observed in the categorisation task. Both the (Unrelated Categorisation > Related Categorisation) ∩ (Categorisation > Baseline) contrast, and the reverse (Related Categorisation > Unrelated Categorisation) ∩ (Categorisation > Baseline) contrast did not reveal any areas, nor did any areas show up in the simple contrast (Unrelated Categorisation > Related Categorisation) or the reverse (Related Categorisation > Unrelated Categorisation).

For the control task, the (Unrelated Central > Related Central) ∩ (Central > Baseline) contrast revealed bilateral anterior superior frontal gyri (SFG), as well as the posterior part of the left middle occipital gyrus. The reverse contrast, (Related Central > Unrelated Central) ∩ (Central > Baseline) activated the bilateral anterior parts of the insulae, bilateral MFG, bilateral medial frontal gyri, and some parts of bilateral inferior temporal cortex (see figure 3C and 3D).

### Random effects analysis

The random effects (RFX) ANCOVA with factors task (4) and relatedness (2) revealed a significant effect of task in bilateral inferior parietal lobules, the bilateral inferior temporal gyri, the left posterior MTG, the bilateral insulae, bilateral MFG and IFG, bilateral superior frontal cortex, and the left medial frontal gyrus (p < .0005) (see figure 2B). Post-hoc analyses showed that for the left-hemispheric areas, the linguistic tasks led to a higher activation than the control task, and for right-hemispheric areas the control task led to a higher activation than the linguistic tasks.

A main effect of relatedness was observed in the right inferior parietal lobule and at the most anterior tip of the right STG (p < .02). No other areas showed a significant relatedness effect in the RFX ANCOVA. An interaction between task and relatedness was observed in the left insula, the medial part of the right STG, and the right orbitofrontal cortex (p < .05).

ANCOVA contrast maps of the relatedness effects within each task revealed that for the control task the left anterior insula was more active in related than unrelated conditions (p < .005). For the offset task, the medial part of the right middle and superior temporal gyrus was more active in unrelated than related conditions. No relatedness effects were observed in the onset and semantic task.

Since we observed a relatedness effect in the fixed effects (FFX) GLM in the expected left posterior middle and STS/STG and the left IFG, but did not find these areas in the RFX ANCOVA, we decided to investigate these areas with a post-hoc RFX ROI analysis. This revealed that the relatedness effect in left IFG in the offset task was consistent across participants (t (13) = 2.20, p = .046). None of the other relatedness contrasts approached significance (all p-values > .3). Similarly, in the area encompassing the posterior STS/STG, a significant relatedness effect was observed for the offset task (t (13) = 2.62, p = .021). None of the other relatedness contrasts approached significance (all p-values > .3).

### Visual areas

Ventral visual areas V1, V2, VP and V4 were localised for each of the participants by means of polar mapping runs. In addition, the Lateral Occipital Complex (LOC) was localised with a separate run in which objects were contrasted with scrambled objects. Participant-specific Region Of Interest (ROI) analyses (random effects model) revealed that, compared to baseline, areas V2, VP, V4 and LOC were consistently activated by all the tasks across participants (all p-values < .01, t (13)). Area V1 did not exhibit a significant activation increase compared to baseline (p > .05 for each task). A significant effect of task was detected in LOC (F (3, 39) = 3.82, p = .017), where the linguistic tasks led to a higher activation than the control task (t (13) = 2.45, p = .029). Significant task effects were not observed in any of the other visual areas (all p-values > .4). In addition, the RFX ROI ANCOVAs did not reveal any effects of relatedness in any of the mapped visual areas, nor were any significant interactions observed between relatedness and task (all p-values > .3).

Possibly, any effects of our experimental manipulations in visual areas were obscured by the procedure of looking at the area as a whole, whereas only a subset of vertices reflected stimulated areas in the visual field. We therefore repeated the analyses with the subset of vertices of an area that was activated in the linguistic tasks (compared to baseline). Again, ROIs were defined separately for each participant. This analysis revealed that for each participant, part of each of the ventral visual areas was activated by the stimuli in the linguistic task. Task effects were observed in areas V2, VP, V4 and LOC (all p-values < .02), but not in V1 (p > .1). Post-hoc contrasts revealed that in the areas in which task effects were observed, the linguistic tasks led to a higher activation than the control task (all p-values < .02, t (13)). No relatedness effects were observed in any of these areas, nor were there any interactions between relatedness and task (all p-values > .3). A similar analysis based on the subset of vertices that was activated by the control task did not reveal any significant main effects or interactions in any of the areas.

## Discussion

The main goal of the present study was to investigate whether cortical areas beyond visual cortex have active representations of the phonological properties of stimuli that are to be ignored by participants. While lying in the scanner, participants viewed two simultaneously presented stimuli at peripheral locations. Meanwhile, a task was performed either at fixation (i.e. ignore pictures), or involving one of the pictures. In half of the blocks' the names of the two pictures were phonologically related to each other, in the other half they were unrelated. Whereas indications of mental representations of ignored stimuli have previously been observed in higher-order visual areas such as LOC [21], inferior temporal cortex [22] and PPA [20], the present study is amongst the first to show effects of distractor processing in cortical areas involved in phonological processing, such as STS/STG, posterior IFG and the insula.

### Relatedness effects

We expected to observe relatedness effects during phonological tasks in areas that are involved in phonological processing, such as the left posterior IFG, left posterior STS/STG, and the left insula. In line with these expectations, unrelated conditions led to higher activations than related conditions in left IFG and STS/STG, when participants were performing the offset-monitoring task. These effects were significant in the FFX analysis, and confirmed by an RFX ROI analysis. These results corroborate and extend earlier findings by de Zubicaray et al. [45], who observed similar effects in STG in a picture-word interference paradigm. They too, observed higher BOLD-signal changes when participants named pictures while ignoring superimposed unrelated words, compared to related words. In addition, we observed such effects in posterior IFG, which has also been implicated in phonological processing. It is often assumed that words are automatically processed or that they capture attention briefly even if they are supposed to be ignored [54]. The present study is the first to show effects of ignored picture processing in an fMRI paradigm. Our findings suggest that, under certain task conditions, distractor pictures that are to be ignored may also activate their phonological codes, implying that they pass through the attentional filter in visual cortex. In fact, distractor processing does not stop at visual processing areas, but proceeds well into the language system. In addition, the systematic variation of phonological relatedness and the observed effects clearly showed that the phonological codes of the distractor picture names were activated.

Interestingly, we did not observe any significant relatedness effects in the onset- but a robust effect in the offset-monitoring task. Possibly, the absence of the onset effect can be explained by the fact that in order to perform this task, the generation of only the first letter of a picture's name is required. Some authors have proposed that the level of stimulus encoding depends on task demands [49] and that the level of processing of ignored stimuli depends on the level or representation required by task demands [30]. The production of a word's phonological code is believed to occur in an incremental manner, that is, from left to right [55,56]. Since the onset task requires only the generation of the first phoneme, participants may not be monitoring letters after the first one. They may be involved in error-monitoring, or preparing for the next trial, which would divert processing resources and attention away from our phonological manipulation, explaining the absence of the relatedness effect in the onset task, both in the behavioural and the fMRI data.

The offset-monitoring task, on the other hand, requires the entire name of a picture to be activated and monitored in order to identify the last phoneme. In this case, the name of the picture, including all phonemes in which overlap with the distractor name exists, are more thoroughly processed, which may lead to an increased detection rate of the relatedness manipulation by phonological processing areas. This interpretation supports the idea that the phonological properties of distractor items are only activated, or at least their overlap with that of the target item only detected, when the task involves scrutinous monitoring of those properties [30]. This idea is further supported by the fact that no phonological relatedness effects were observed in IFG in the semantic judgment task, in which no explicit naming of the target picture is required. Similarly, a recent ERP study showed that semantic and phonological priming effects can be observed if participants were involved in an explicit naming task, but that only the semantic effects prevailed in a non-linguistic, natural size-judgment task [57].

Unexpectedly, we also observed a phonological relatedness effect in the control task (bar-width comparison). This effect was primarily observed in the anterior parts of the left insula. Importantly, the relatedness effect in this area is reversed compared to the one observed in the offset task. That is, blocks with phonological related picture pairs led to higher activation than unrelated blocks. These findings cannot be explained in terms of stimulus differences between related and unrelated conditions for two reasons. First, each picture was repeated equally often in related and unrelated conditions and at the same position. Second, since the exact same picture combinations were used across the tasks, any effects of the pairing of the stimuli should also have appeared in the linguistic tasks. We will discuss the finding of a relatedness effect in light of insular functions in the section below.

Interestingly, no behavioural effects of relatedness were observed in the current study, which is at odds with previous picture-picture interference studies in which facilitation effects were observed in trials where the names of the pictures were phonologically related instead of unrelated [37-39]. The fact that we did not find any behavioural effects may indicate that our paradigm may not be sensitive enough to capture phonological facilitation effects. One reason for the lower sensitivity of our study may be the use of the phoneme monitoring task, which is more unnatural than e.g. a naming task. It involves direct attention to the sequence of single phonemes [58] rather than to the entire string, and requires a different response (push-button rather than naming) and hence may be less sensitive to phonological relatedness effects. It should also be noted that the amount of trials in which a response was needed was very low (i.e. 2 per block), perhaps too low to reliably estimate response time differences.

Alternatively, it cannot be excluded that behavioural effect were absent because the name of the distractor stimulus was accessed after task execution. Since stimuli were not masked, covert attention shifts to the distractor picture may have occurred *after *target identification. In that case, the neural modulations observed in some of the related conditions would be caused not by an interaction of phonological codes of simultaneously identified stimuli, but by the residual activation of the phonological code of the target when the distractor is subsequently identified. Presumably, such a shift would not occur before enough information is present to prepare a response to the current task. Phoneme monitoring is believed to take place after internal syllabification of the word [58], which is completed around 450 ms after picture onset [40]. Hence, the *earliest *time in which attention might be shifted to the distractor stimulus is around 450 ms, approximately 200 ms after stimulus offset. We believe that the actual limit is even higher, given the peripheral location of our stimuli, and the fact that they occupy separate locations. Hence, covert, spatial attention shifts would be required, which take time to prepare and execute [59]. Given the short stimulus duration and the rapid decay of information in the iconic store [60,61], this leaves a very short interval in which the distractor stimulus must be identified.

### Hemispheric lateralisation and task-specific activations

In line with previous observations that in right-handed people, language processing is lateralised mainly towards the left hemisphere [40,62], the linguistic tasks activated an extensive, left-hemispheric network compared to the control task. This network included the left MTG (semantic processing) and STS/STG (phonological processing), and the left insula, IFG and MFG (semantic/phonological processing).

It has been suggested in the literature that the IFG plays a role in both phonological and semantic processing, but that the anterior part (BA 45/47) is specialised in semantic processing, whereas the posterior part (BA 44/45) plays an important role in phonological processing [40-44,63]. Corroborating previous studies, we observed that phoneme monitoring activated the posterior part of IFG stronger than semantic categorisation, whereas the reverse was true for the anterior part of the IFG. Noesselt et al. [41] proposed that the posterior part of IFG may be specialised for phonological processing, and that this is automatically activated in semantic tasks. In line with this proposition, we observed a significant activation increase in this area in the semantic task compared to the control task, indicating that phonological processing was taking place during semantic categorisation. However, the fact that this activation is consistently lower than that in the phonological task indicates that perhaps phonological processing is not as thorough in a semantic categorisation task, which could explain the absence of a relatedness effect in this condition. In addition, the semantic task significantly activated a large portion of the MTG stronger than the phonological task. MTG has been associated with the storage of long-term semantic memory, whereas the anterior IFG is believed to involve selection and control from that memory [43,50]. Therefore, although these areas are activated in phonological tasks (semantic retrieval takes place before the phonological code is activated [64,65], the semantic task requires an active judgement of the category membership of the picture. This requires stronger selective processes, leading to the higher activation of these areas.

The role of the insula in language processing is less clear. Insular regions have been associated with several kinds of aphasia, including Broca's and Wernicke's aphasia, indicating its role in phonological processing. The anterior part of the insula was recently found to be susceptible to sub-lexical spelling-to-sound processing [66], which would explain the higher activation in the phonological tasks compared to the semantic task. However, in the present study, the insula was just as active in the control task as in the phonological tasks. In addition, a phonological relatedness effect was observed in this area, which implies that the names of both pictures were phonologically processed in the control task. Related picture pairs were associated with higher activation levels than unrelated ones (i.e. the reverse pattern from that observed in the IFG). Interestingly, similar results were reported in a recent study in which successively presented pictures or words had to be named or read, which could be phonologically related or unrelated [67]. The observation that insula was activated more strongly if two successive words/pictures were related than when they were unrelated, closely fits the data pattern observed in the current study, and led these authors to conclude that the insula might be involved in discriminating competing phonological codes.

It is unclear, however, why we observed this effect in the control task, but not in the linguistic tasks. Perhaps the control task (bar-width discrimination) was not attentionally demanding enough to fully engage participants' attention. Residual resources may have been employed to process the pictures, while the insula monitored the results of this attentional spill-over. It is also unclear, why the direction of the effect is reversed compared to the effect we observed in the IFG in the offset task and why the insula is insensitive for phonological relatedness in that condition. The absence of phonological relatedness effects in the insula in linguistic tasks implies that this monitoring only took place in non-linguistic tasks (perhaps scanning the environment for stimuli with linguistic content) or when task demands allowed additional resources to be diverted to distractor processing. In the insula, related picture pairs were associated with higher activation levels than unrelated ones (i.e. the reverse pattern from that observed in the IFG). This implies that perhaps one feature of this monitoring process is the detection of salient features of linguistic stimuli, in this case the phonological relatedness between two peripherally presented stimuli. In this respect, it would be interesting to observe whether the insula is also involved in detecting salient stimuli in an unattended auditory stream, as occurs the cocktail party effect [13,14]. However, due to the unexpected observation of relatedness effects in the insula, the absence of such effects in the linguistic tasks, and the poorly understood role of the insula in language processing, additional research is required to elucidate its role in the processing and monitoring of ignored stimuli.

## Conclusion

This study is the first to show that phonological properties of ignored pictures can be processed by phonological processing areas such as Wernicke's area and the posterior IFG. These results implicate that ignored pictures can be identified beyond their physical properties. Since effects of phonological relatedness were not observed in all task conditions, we suggest that the properties of a picture that are identified depend on task demands and depth of processing. Whereas we addressed the identification of phonological properties of ignored pictures, future studies may address relatedness effects with other stimulus features, such as semantics.

## Methods

### Participants

Fourteen volunteers (6 Females, age 20–31 years) participated in this study. All were right-handed, neurologically healthy, native speakers of Dutch, had normal or corrected-to-normal visual acuity and were paid for their time. This research project was approved by the Ethics Committee Psychology (ECP) of Maastricht University and written informed consent was obtained from each participant prior to the scanning session.

### Stimuli

Twenty-four pairs of white line drawings of common objects on a black background were used as stimuli in this experiment. The Dutch names of the pictures within a pair were phonologically related, i.e. they shared at least the same consonant-vowel onset structure with each other, e.g. "VOS" (fox), "VORK" (fork) (average word length 5 letters, average onset overlap 2.2 letters). The stimuli were divided into three sets of eight pairs, encompassing the stimuli for the 'related' blocks. The same sets were recombined into 'unrelated' sets, so that the members were phonologically and semantically unrelated and did not have offset overlap. One member of each pair was designated as an attended picture (i.e. occurring at the location closest to the fixation point), and one as a distractor picture (occurring at the location furthest away from the fixation point). Within each set of eight attended pictures, two targets were designated per task (i.e. required an overt response). E.g. a set of eight attended pictures might have two pictures starting with an 'r' (onset task), two ending with a 'g' (offset task), and two buildings (categorisation task) (see appendix). Target trials in the fourth task (bar-width discrimination) had the same picture pairs in the display as the three other tasks but were defined based on the properties of the bars at the centre of the screen. Each set of 'attended' pictures was presented eight times: once for each of the four tasks both in phonologically related and in unrelated conditions. Hence, visual stimulation and amount of targets per block across these tasks was identical; the only parameter that differed was task instruction.

In related blocks, an 'attended' picture was presented with the distractor picture that was phonologically related to it. Unrelated blocks were created by pairing 'attended' pictures of one set with the distractor pictures of another set. This insured that these new combinations were phonologically unrelated. Care was taken to avoid any semantic relationship or offset overlap within unrelated pairs. Each picture that appeared at a distractor position in a related block, appeared at the same position, but paired with a different 'attended' picture in an unrelated block. The same holds for 'attended' pictures. Each of these sets was presented 4 times per fMRI run: once per task (onset monitoring, offset monitoring, semantic judgement, bar-width discrimination). This way, it was assured that across tasks visual stimulation was identical. In addition, the number of targets across blocks was kept constant and targets never occurred at distractor locations.

Distractor pictures never matched target criteria for a certain condition, were always semantically unrelated to the 'attended' picture, and had a different name offset. In related conditions, they shared the initial consonant vowel structure with the target. In unrelated conditions, they did not share onset phonemes with the target.

### Procedure

Trials were presented in a block-design. An fMRI run started with the presentation of a blank screen for 18 seconds, followed by an instruction centred on the screen for 1 second, consisting of two keywords indicating the target location and the target. Subsequently, another blank screen followed for 1 second, after which a block started. Blocks consisted of the presentation of eight trials of two stimuli simultaneously presented above each other in the upper right quadrant of the screen for 250 ms, followed by a blank screen for 1250 ms, after which a new pair of stimuli was presented. The 'attended' picture was centred at 2.5° from the fixation cross, the distractor stimulus 2° degrees above it. Each picture subtended approximately 1.5° degrees of visual angle.

During stimulus presentation, participants performed one of four tasks, indicated to them prior to the start of the block. In the onset monitoring task, participants were asked to judge whether the name of the picture closest to the fixation point started with a certain letter. The offset monitoring task was similar, but involved the decision whether the picture name ended with a certain letter. The semantic task involved a category judgment in which participants responded to pictures belonging to a certain category (e.g. mammals, musical instruments). The control task involved a bar-width discrimination task: participants were asked to attend the two bars (.2° in height) that replaced the fixation cross. These bars were presented .15° degrees above and below the fixation cross. The width of the bars varied between 1 and 5 pixels, and the difference between the bars was never more than 1 pixel. A button press was required in case the bars were of the same width (2 targets per block). Participants were instructed to fixate the centre of the screen (fixation cross) throughout the experiment and to minimise head and eye-movements while in the scanner. In a previous ERP pilot experiment involving a similar design, eye movements were monitored. These revealed that participants were able to maintain fixation across conditions. Furthermore, no differences were observed between related and unrelated conditions, excluding poor fixation as an explanation for any relatedness effect. Participants were not made aware of the phonological relationship between the stimuli and were asked to ignore the distractor stimuli.

The order of the blocks was randomised for each run and each participant. Random permutations of the eight conditions were repeated three times per run, with the restriction that the same task never appeared twice in succession.

### Scanning parameters

Images were acquired on a 3T Siemens Allegra head scanner (Siemens Medical Systems, Erlangen, Germany) using a standard head coil. Thirty-two oblique axial slices (in-plane resolution: 3.5 × 3.5 mm, slice thickness: 3.5 mm, interslice distance 0 mm) covering the entire cortical volume were acquired using an echo planar imaging sequence (TR = 2000 ms, TE = 29 ms, matrix size: 64 × 64). There were 268 volumes per run, the first 2 of which were skipped due to the T1 saturation effect. Functional slices of each run were aligned to a high resolution (voxel size 1 × 1 × 1 mm^3^) anatomical dataset acquired using a T1-weighted 3D MP-RAGE (magnetization-prepared rapid acquisition gradient echo) sequence (192 sagittal slices, TR = 2.3 s, TE = 3.93 ms).

The participants were placed comfortably in the scanner and their head was stabilised with foam pads in order to reduce head motion. Mounted on the head coil was a mirror through which they could see the stimuli projected on a screen. Stimulus presentation was synchronised with MR data acquisition by triggering the stimulus program with the first MR pulse.

### Region of interest localisation

Polar maps were acquired using a rotating, red-green blocked wedge of 33.75 degree polar angle covering eccentricities from 1° to 17° of visual angle. Wedges were filled with a checkerboard pattern of red and green squares that reversed polarity 8 times per second and did a full rotation within 64 seconds. Thus, each pixel in a circular field of view was activated every 64 seconds for a duration of 6 seconds. A functional run took 552 seconds, i.e. 8 cycles of rotating wedges plus 20 seconds lead in and lead out time. Borders between areas were defined based on the alternating vertical and horizontal meridians that demarcate the borders of these areas [51,68]. Ventral visual areas V1, V2, VP and V4 were defined for each participant. Area TEO could not be reliably located for most participants by this method, but was defined as the area anterior to V4 that was activated reliably by the stimuli across participants, and was located at comparable Talairach coordinates as in previous studies [5,10].

The LOC was localised by contrasting blocks of objects with blocks of scrambled objects (FDR-corrected, q = .05). Each participant viewed 2 runs of this localiser which contained 3 blocks of objects and 3 blocks of scrambled objects, lasting 20 s each, separated by 15 s baseline intervals. Scrambled objects were created by dividing each object picture in a 20 by 20 square grid and randomly rearranging the grid elements.

Scanning parameters were the same for the localiser runs and the experimental runs. Pre-processing procedures were also identical, except that no spatial smoothing was applied.

### Analysis

Data were analysed using the BrainVoyager QX package (Brain Innovation, Maastricht, the Netherlands). Functional data were corrected for motion in three dimensions, slice scan-time corrected and spatially smoothed with a 4 mm FWHM Gaussian kernel. Subsequently, linear drifts were removed from the signal and data were high-pass filtered to remove slow frequency drifts up to 3 cycles per time course. After pre-processing, functional data were aligned to the high-resolution anatomical images, morphed to fit Talairach dimensions [69] and combined into a 4-dimensional dataset for each run and each participant.

In order to improve anatomical correspondence between participants, all individual brains were segmented at the grey/white matter boundary (using a semi-automatic procedure based on intensity values) and the reconstructed cortices were aligned using curvature information reflecting the gyral/sulcal folding pattern. The reconstructed folded cortical representations of each participant and hemisphere were morphed into a spherical representation, each vertex on the sphere (spherical coordinate system) corresponding to a vertex of the folded cortex (Cartesian coordinate system) and vice versa. The curvature information computed in the folded representation was preserved as a curvature map on the spherical representation, driving inter-cortex alignment and minimizing the mean squared differences between the curvature of a source sphere and the average of all spheres. Visual inspection and a measure of the averaged mean squared curvature difference revealed that the alignment of major gyri and sulci was achieved reliably by this method.

The established correspondence mapping between vertices of the cortices was used to align the time courses for multi-subject General Linear Model data analysis. The GLM included eight predictors: onset related, onset unrelated, offset related, offset unrelated, category related, category unrelated, control related, and control unrelated. FFX effects statistical maps were FDR-corrected [70] at an FDR level of .05. Second level analysis used the average of the estimated beta weight values of each participant and condition from the FFX analysis, which were then included in a RFX ANCOVA model with factors task (4 levels) and relatedness (2 levels). Reported RFX ANCOVA maps were uncorrected for multiple comparisons. For each participant, functionally defined regions of interest (ROI) were specified based on the localiser runs. The activation in these areas was analysed with an RFX ROI approach, using functional data from each individually defined ROI.

## Authors' contributions

MB participated in the design of the study, acquired and analysed the data and participated in the draft of the manuscript. BMJ conceived the study and participated in the design of the study, the analysis of the data and the draft of the manuscript. All authors read and approved the final manuscript.

## Appendix A

List of related word pairs. Unrelated pairs were formed by recombining related pairs. See text for details.

1. banaan – ballon

2. bord – bom

3. fabriek – fagot

4. fazant – fakkel

5. galg – gans

6. gitaar – gieter

7. hamster – harpoen

8. hand – hark

9. kaas – kaart

10. kerk – kers

11. ladder – lasso

12. liniaal – libel

13. map – maïs

14. matras – masker

15. pion – pistool

16. paraplu – paprika

17. raket – radio

18. rok – rolstoel

19. tong – tor

20. trap – tram

21. wieg – wiel

22. worst – wolk

23. vos – vork

24. vulkaan – vulpen
